# Swallowing and feeding after disease-modifying treatment for spinal muscular atrophy: a systematic review of assessment modalities and outcomes

**DOI:** 10.1186/s13023-025-04118-z

**Published:** 2026-01-08

**Authors:** Yasmina Martí, Ksenija Gorni, Sandhya Kumari, Anadi Mahajan, Giovanni Baranello, Liesbeth De Waele, Katlyn E. McGrattan

**Affiliations:** 1 F. Hoffmann-La Roche Ltd, Basel, Switzerland; 2 Bridge Medical Consulting Ltd, Richmond, London, UK; 3 The Dubowitz Neuromuscular Centre, National Institute for Health Research Great Ormond Street Hospital Biomedical Research Centre, Great Ormond Street Institute of Child Health University College London, and Great Ormond Street Hospital Trust, London, UK; 4 NMRC for Children, Department of Paediatrics, University Hospitals Leuven, Leuven, Belgium; 5 Department of Development and Regeneration, KU Leuven, Leuven, Belgium; 6 Department of Speech-Language-Hearing Science, University of Minnesota, Minneapolis, Mn USA; 7 Department of Rehabilitation, Masonic Children’s Hospital, Minneapolis, Mn USA

**Keywords:** Bulbar function, Feeding, Nusinersen, Onasemnogene abeparvovec, Risdiplam, Spinal muscular atrophy, Swallowing, Systematic literature review

## Abstract

**Background:**

Feeding and swallowing deficits are reported across the spectrum of spinal muscular atrophy (SMA), with more profound symptoms associated with more severe disease. Patients treated with disease-modifying therapies (DMTs) demonstrate significantly improved life expectancy and motor function relative to untreated counterparts; however, limited data exist regarding the impact of DMTs on bulbar integrity, with evidence suggesting bulbar symptoms may persist, even when motor function has improved. This systematic literature review was conducted to identify assessments used to evaluate swallowing in patients with SMA treated with DMTs, and to describe the impact of DMTs on swallowing and feeding outcomes. Embase, MEDLINE, and Cochrane central were searched from May 2021 to February 2024 for studies reporting swallowing and feeding outcomes in patients treated with nusinersen, onasemnogene abeparvovec, and risdiplam.

**Results:**

Seventy-one studies were included. The majority of studies (99%, *n* = 71) reported functional swallow outcomes, such as oral intake status or patient-reported outcomes, and only 19% reported results from clinician-administered assessments. Results from imaging assessments were rarely reported (5%, *n* = 4 studies). Only 68% of studies reported results from both pre- and post-treatment assessments. Patients who received DMT prior to symptom onset were found to have good functional outcomes, with 84–100% receiving full oral nutrition. Treatment after symptom onset yielded variable results, with trends suggesting that treatment outcomes are influenced by the level of impairment at baseline and the type of swallowing assessment used. The ability to maintain pre-treatment swallow integrity was variable across studies.

**Conclusion:**

Although evidence suggests that DMTs can preserve or improve bulbar function in SMA, swallowing and feeding are not regularly or homogeneously assessed across the literature, making comparisons across studies difficult. Standardised and validated assessments of swallowing physiology and swallowing function are needed to understand what factors may influence bulbar outcomes with DMTs.

**Supplementary Information:**

The online version contains supplementary material available at 10.1186/s13023-025-04118-z.

## Background

Spinal muscular atrophy (SMA) is an autosomal recessive, progressive neuromuscular disease with an estimated global incidence of ~ 1 in 10,000 live births [1]. SMA is characterised by a deficiency in survival of motor neuron (SMN) protein [2], which leads to progressive muscle denervation, skeletal muscle atrophy, muscle weakness, and loss of motor function [1]. Although the severity of these deficits can vary greatly, feeding and swallowing deficits have been reported across all phenotypes [3–6].

In the most severe form, Type 1 SMA, bulbar deficits are prevalent, with infants exhibiting a rapid degeneration of oropharyngeal sucking and swallowing physiology that impedes the infants’ ability to express milk from the nipple, close the laryngeal opening to prevent aspiration, and clear ingested material from the pharynx [7]. The cumulative effect of these deficits in untreated patients has historically resulted in the reliance on tube nutritional support by 12 months of age [8], with children at high risk of developing aspiration pneumonia [4, 9]. In milder phenotypes (Types 2 and 3 SMA), reported deficits are isolated to chewing, instances of choking, and fatigue with meals [5, 6].

Given the prevalence and significance of these deficits, assessment of oropharyngeal swallowing integrity is critical in patients with SMA. Functional swallowing assessments can be categorised into two types: (1) clinician-administered assessments and (2) patient-reported outcomes. Clinician-administered assessments are those in which a provider makes determinations about the integrity of an individual’s swallowing biomechanics. One type of clinician-administered assessment is the clinical swallow evaluation, during which a clinician makes determinations about swallowing integrity using visual observation as they watch an individual eat and drink. Though valuable, clinical swallow evaluations are noted to have poor sensitivity for detecting pharyngeal swallow impairments, which cannot accurately be identified via observation alone [10–12]. As such, clinicians rely largely on findings from another form of clinician-administered assessment, an instrumental swallowing assessment, to fully characterise the integrity of internal biomechanics not visible to the naked eye. The gold standard in instrumental swallowing assessment is the videofluoroscopic swallow study, a procedure that uses an x-ray to quantify the integrity of oropharyngeal swallowing physiology and the impact on bolus flow, such as penetration or aspiration [13]. Other instrumental swallowing assessments such as fibreoptic endoscopic evaluation of swallowing and high-resolution manometry can also provide valuable insights into the integrity of these internal processes.

Whilst measures of swallowing integrity are critical, assessment of the functional implications of these biomechanics are of equal importance. Functional swallow outcomes can be reported in a variety of ways, including descriptive assessments, oral intake status, and the perception of swallowing integrity collected through patient-reported outcomes.

Disease-modifying therapies (DMTs) designed to halt disease progression hold promise for facilitating swallowing integrity. These treatments, including nusinersen (SPINRAZA^®^), onasemnogene abeparvovec (ZOLGENSMA^®^), and risdiplam (EVRYSDI^®^), have been shown to significantly improve the life expectancy and gross motor integrity of patients with SMA when compared with their untreated counterparts [14]. However, there are limited data regarding the impact of DMTs on bulbar integrity, with some evidence suggesting that bulbar symptoms may persist in patients even when motor function has improved [15].

Given the implications in validity of outcomes, the aim of this systematic literature review (SLR) was to identify the types of swallowing assessments used in patients with SMA treated with DMTs, and to describe the impact of DMTs on swallowing and feeding outcomes in SMA.

## Methods

This SLR was reported in accordance with PRISMA guidelines. Searches were conducted in the Embase^®^, MEDLINE^®,^ and Cochrane central electronic databases and included studies published from database inception to the end of February 2024. A search strategy was developed based on the Population, Interventions, Comparison, Outcomes, and Study (PICOS) design framework [16] (Supplementary Tables 1 & 2, Additional File 1). Eligible publications included randomised control trials (RCTs), non-RCTs, single-arm studies, and real-world observational studies (prospective and retrospective) that reported data from patients with presymptomatic and symptomatic Types 1–3 SMA who were treated with at least one SMA DMT (nusinersen, onasemnogene abeparvovec, or risdiplam). Cross-sectional studies and case reports/series were excluded. Supplementary searches were performed to identify data published as relevant conference proceedings (see Supplementary Table 3, Additional File 1 for a full list of conferences included in the supplementary searches).

Assessment of bias and quality of evidence of the included studies was conducted using the National Institute for Health and Care Excellence (NICE) manufacturer’s template for RCTs [17], the Agency for Healthcare Research Quality (AHRQ) checklist for non-RCTs and single-arm trials [18], and the Newcastle-Ottawa Scale for observational studies [19].

Data were extracted from the individual studies and summarised qualitatively. Outcomes were classified as clinician-administered swallowing assessments (clinical swallow evaluation and instrumental swallow evaluation) and functional swallowing outcomes (oral intake status and patient-/caregiver-reported outcomes) for descriptive analysis.

## Results

**Fig. 1:**
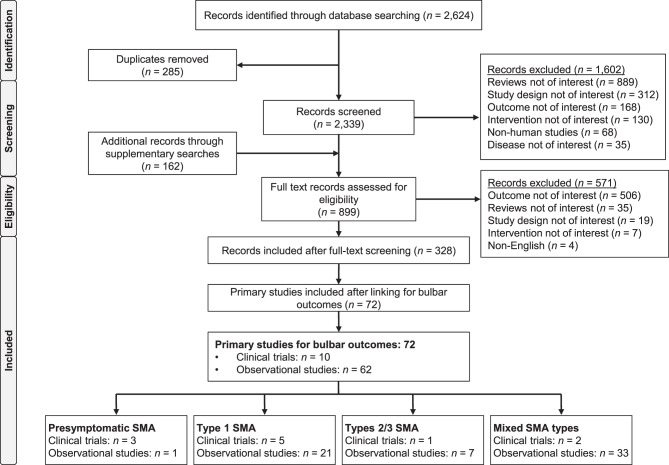
PRISMA diagram. Abbreviations: PRISMA, Preferred Reporting Items for Systematic reviews and Meta-Analyses; SMA, spinal muscular atrophy

A total of 2,624 records were identified through the SLR and 162 records were identified via supplementary searches (PRISMA diagram – Fig. 1). After screening for eligibility, 72 primary studies (10 clinical trials [14%] and 62 observational studies [86%]) were identified that reported swallowing and feeding outcomes in patients with SMA treated with DMTs. Three clinical trials and one observational study reported data in presymptomatic SMA, 26 studies (five clinical trials, 21 observational studies) reported data in a Type 1 SMA population, eight studies reported data from a Type 2/3 SMA population (one clinical trial and seven observational studies), and 35 studies (two clinical trials and 33 observational studies) reported data from mixed SMA type populations. Only 48 (68%) investigations reported pre-treatment and post-treatment assessment of bulbar function.

We have classified the results into studies that reported on clinician-administered assessment (clinical swallow evaluation and instrumental assessments) and studies that reported on functional swallow outcomes. Out of 72 studies identified, almost all (71/72, 99%) reported functional swallowing outcomes, such as oral intake status (61/72, 85%) and patient-reported outcomes (14/72, 19%). Only 14 (19%) studies used clinician-administered assessments; most of these studies (11/14, 79%) reported results from clinical swallow evaluations, with only 5/14 (36%) using instrumental assessments. Results were reported using descriptive statistics for almost all (67/71, 94%) investigations.

### Clinician-administered swallowing assessments

#### Clinical swallow evaluation

Twelve publications from 11 studies reported on swallowing outcomes following a DMT using clinical swallow evaluation (Table 1).

**Table 1:** Observational clinical swallow evaluation

DMT	Study [reference]	Study type	*N*	SMA type	*SMN2* **copy number**	Age at onset Median (range/IQR) Mean [range/SD]	Age at treatment initiation Median (range/IQR) Mean [range/SD]	Outcome assessed	**At timepoint of interest (%)**^a^					
									Baseline	6 months	12 months	18 months	24 months	Other (timepoint)
**Studies in presymptomatic SMA**														
Nusinersen	*NURTURE* *De Vivo 2019* [20]	*SA*	*15*	*Presymptomatic*	*2*	*NR*	*19.0d (8–41)* *19.5d [9.29]*	*Good sucking and swallowing (HINE-1 score 3)*	*-*	*-*	*-*	*-*	*80* *(Day 778)*	*-*
			*10*		*3*	*NR*	*23.0d (3–42)* *22.3d [12.45]*	*Good sucking and swallowing (HINE-1 score 3)*	*-*	*-*	*-*	*-*	*100* *(Day 778)*	*-*
Onasemnogene abeparvovec	*SPR1NT* *Strauss 2022* [21]	*SA*	*14*	*Presymptomatic*	*2*	*NR*	*21.0d* *(8.0–34.0)* *20.6d* *[SD: 7.9]*	*Normal swallowing * *of thin liquids*^b^	*100*	*-*	*-*	*93*	*-*	*-*
	*SPR1NT* *Post-hoc analysis* *Shell 2023* [22]		*14*		*2*	*NR*	*20.6d* *(8.0–34.0)*	*Normal swallowing * *of thin liquids*^b^	*-*	*-*	*-*	*100*	*-*	*-*
			*15*		*3*	*NR*	*28.7d* *(9.0–43.0)*	*Normal swallowing* *of thin liquids*^b^	*100*	*-*	*-*	*-*	*100*	*-*
Risdiplam	*RAINBOWFISH Finkel 2022* [23]	*SA*	*7*	*Presymptomatic*	*2,3, and ≥4*	*-*	*26.5d* *(16–40)* *(n = 18)*	*Able to swallow*	*100*	*-*	*100*	*-*	*-*	*-*
**Studies in a Type 1 SMA population**														
Nusinersen	De Lucia 2020 [24]	ROS	23	Type 1	2, 3	NR	NR	Oral disorders (swallow’s disease and/or difficulties chewing)	*30*	*-*	*-*	*70*	*-*	*-*
Onasemnogene abeparvovec	*START* *McGrattan 2023* [25]	*DC/DE*	*11*	*Type 1*	*2*	*NR*	*NR*	*Swallowing normally*	*36*	*-*	*-*	*-*	*100*	*-*
	*STR1VE-EU* *Mercuri 2021* [26]	*SA*	*33*	*Type 1*	*2*	*1.6m* *[SD: 0.9]*	*4.1m* *[SD: 1.3]*	*Intact swallowing*^c^	*97*	*-*	*-*	*-*	*-*	*-*
	*STR1VE-EU* *McGrattan 2023* [25]	*SA*	*33*	*Type 1*	*2*	*1.6m* *[SD: 0.9]*	*4.1m* *[SD: 1.3]*	*Swallowing normally*	*97*	*-*	*-*	*91*	*-*	
	*STR1VE-US* *Day 2021* [27]	*SA*	*22*	*Type 1*	*2*	*1.8m* *(1.0–3.0)* *1.9m* *[SD: 1.2]*	*3.5m* *(2.7–5.3)* *3.7m* *[SD: 1.6]*	*Swallowing normally*^c^	*100*	*-*	*-*	*55 (at 18m of age)*	*-*	*-*
								*Normal or functional swallow with consistencies other than thin liquid*	*-*	*-*	*-*	*32 (at 18m of age)*	*-*	*-*
	*STR1VE-US* *McGrattan 2023* [25]	*SA*	*22*	*Type 1*	*2*	*1.8m* *(1.0–3.0)* *1.9m* *[SD: 1.2]*	*3.5m* *(2.7–5.3)* *3.7m* *[SD: 1.6]*	*Swallowing normally*	100	-	-	91	-	-
	*McGrattan 2023* [25] *START* *STRIVE-EU* *STR1VE-US*	*Post-hoc analysis*	65	Type 1	2	*NR*	*NR*	*Normal* *swallow*	88	-	-	-	-	92 (end of study)^c^
Risdiplam	*FIREFISH* *Part 2* *Darras 2021* [28]	*SA*	*41*	*Type 1*	*2*	*1.5m* *(1.0–3.0)*	*5.3m* *(2.2–6.9)*	*CGI-C: improvement or no change*	*-*	*-*	*71*	*-*	*-*	*-*
								*CGI-C: very much improved*	*-*	*-*	*7*	*-*	*-*	*-*
								*CGI-C: much improved*	*-*	*-*	*29*	*-*	*-*	*-*
								*CGI-C: minimally improved*	*-*	*-*	*10*	*-*	*-*	*-*
								*CGI-C: unchanged*	*-*	*-*	*24*	*-*	*-*	*-*
								*CGI-C: worse*	*-*	*-*	*10*	*-*	*-*	*-*
								*CGI-C: much worse*	*-*	*-*	*7*	*-*	*-*	*-*
								*CGI-C: minimally worse*	*-*	*-*	*2*	*-*	*-*	*-*
Nusinersen, onasemnogene abeparvovec, risdiplam^d^	Zang 2023 [29]	POS	10	Type 1	2	2.0m (0–7)	3.8m (0.7–8.9)	Weak suck	60.0	-	50.0	-	-	-
								Strong suck	10.0	-	20.0	-	-	-
								Experienced fatigue related to feeding	66.67 *N* = 9	-	37.5 *N* = 8	-	-	-
**Studies with a Type 2/3 SMA population**														
Nusinersen	Ambawatte 2022 [30]	ROS	11	Types 2/3	2–4	NR	NR	Swallowing difficulties	9.1	-	-	-	-	-
Risdiplam	Ambawatte 2022 [30]	ROS	9	Type 2	3,4	NR	NR	Swallowing difficulties	22.2	-	-	-	-	-
**Studies with a mixed SMA type population**														
Nusinersen switch to onasemnogene abeparvovec	AlNaimi 2022 [31]	ROS	11	Type 1/2	2/3	NR	(4–23m)	Swallowing dysfunction	45.5	-	-	-	-	45 (PGT)
			9	Type 1	2	NR	12m (4–23)	Swallowing dysfunction	56	-	-	-	-	-
Onasemnogene abeparvovec			2	Type 2	3	*NR*	*22m* *(21–23)*	Swallowing dysfunction	0	-	-	-	-	-

Abbreviations: CGI-C, Clinical Global Impressions Scale-Clinical change; d, days; DC, dose comparison; DE, dose escalation; DMT, disease-modifying therapy; HINE-1, Hammersmith Infant Neurological Examination, Module 1; IQR, interquartile range; m, months; NR, not reported; PGT, post-gene therapy; POS, prospective observational study; ROS, retrospective observational study; SA, single arm; SD, standard deviation; SMA, spinal muscular atrophy; SMN, survival of motor neuron

^a^ Values correspond to percentages of patients (*N*), unless stated otherwise. Timepoint refers to time on treatment, unless specified

^b^ As demonstrated through a formal swallowing test with thin liquids

^c^ As demonstrated through a formal swallowing test with thin or very thin liquids

^d^ Nine children received onasemnogene abeparvovec; five of these nine had already received nusinersen. One child received risdiplam

Clinical trial publications are in italics

#### Presymptomatic SMA

Promising bulbar outcomes were reported amongst presymptomatic patients across all DMTs (nusinersen, onasemnogene abeparvovec and risdiplam; *n* = 4 studies). Clinical swallow evaluation outcomes amongst infants with two copies of the *SMN2* gene revealed ‘good’ sucking and swallowing in 80% of infants treated with nusinersen [20], ‘normal’ swallowing of thin liquids in 93–100% infants treated with onasemnogene abeparvovec [21, 22], and ‘the ability to swallow’ in 100% of infants treated with risdiplam [23]. Length of follow-up ranged from 18 to 24 months across investigations.

#### Symptomatic SMA

Less optimistic outcomes were found when treatment was initiated after symptom onset for patients with Type 1 SMA. The ability to generalise results across the six studies identified was limited by the variety in reporting methods: three studies (50%) reported proportions of patients who had ‘normal’ or ‘intact’ swallowing, one study reported swallowing stability relative to baseline [28]; one reported sucking strength in infants [29] and one reported the presence of swallowing or chewing dysfunction [24]. The proportion of patients who achieved ‘normal’ swallowing after treatment with nusinersen or onasemnogene abeparvovec varied widely, with 30–100% of patients reported to have ‘normal’ swallowing after at least 18 months of follow-up [24–27]. Relative to baseline, studies indicate the majority of patients ($\ge$60%) maintain or improve their pre-treatment swallow integrity regardless of the DMT they receive (nusinersen, onasemnogene abeparvovec or risdiplam) over 12–24 months of follow-up.

Longitudinal data were not available for feeding and swallowing outcomes in Types 2 and 3 SMA.

### Instrumental swallowing assessments

Five observational studies reported data from instrumental swallowing assessments in patients treated with DMTs (Table 2). Four studies used videofluoroscopic swallow study or fibreoptic endoscopic evaluation of swallowing to measure swallow biomechanics and one study evaluated contracture in the temporomandibular joint by measuring mouth opening. Instrumental investigations that examined swallowing were limited in sample size, did not report biomechanics in a standardised method (only two of the four studies using imaging specified that the penetration–aspiration scale was used to score swallowing integrity [4, 29]), and results were frequently restricted to reports of penetration or aspiration alone. The length of follow-up was also unspecified in 3/5 (60%) studies.

**Table 2:** Instrumental swallowing assessments

DMT	Study [reference]	Study type	*N*	SMA type	*SMN2 ***copy number**	**Age at disease onset** Median (range/IQR) Mean [range/SD]	Age at treatment initiation Median (range/IQR) Mean [range/SD]	Instrumental assessment used	Outcome assessed	**At timepoint of interest**^a^					
										Baseline	6 months	12 months	18 months	24 months	Other (timepoint)
**Studies in presymptomatic SMA**															
Nusinersen switch to onasemnogene abeparvovec	Chiang 2023 [32]	ROS	1	Presymptomatic	2	NR NBS-diagnosed	**Nu:** 6w **OA:** 9w	VFSS	Laryngeal penetration and aspiration with thin liquids	100 (aged 5m)	-	-	-	-	0 (NR^b^)
**Studies in a Type 1 SMA population**															
Nusinersen	van der Heul 2020 [4]	POS	5	Type 1	2	-	-	VFSS	Silent aspiration	-	-	-	-	-	80 (NR)
Nusinersen switch to onasemnogene abeparvovec Onasemnogene abeparvovec Risdiplam^c^	Zang 2023 [29]	POS	10	Type 1	2	2.0m (0.0–7.0)	3.8m (0.7–8.9)	FEES	PAS: median score	5	4.5	-	-	-	-
									No penetration or aspiration (PAS = 1)	10	10	-	-	-	-
									Penetration (PAS = 2–5)	40	50	-	-	-	-
									Aspiration with cough (PAS = 6–8)	40	20	-	-	-	-
									Silent aspiration (PAS = 8)	30	10				
**Studies with a mixed SMA type population**															
Onasemnogene abeparvovec	Beri 2023 [33]	POS	22	NR	NR	NR	NR	-	Mean maximal mouth opening (SD)	18.8 (5.9)	-	-	-	-	20.7 (5.0) (3m)
Onasemnogene abeparvovec and nusinersen switch to onasemnogene abeparvovec^d^	AlNaimi 2022 [31]	ROS	3	Type 1/2	2/3	Type 1: 2m (0.3–6) Type 2: 20m (21–23)	Type 1: 12m (4–23) Type 2: 22m (21–23)	VFSS	Normal swallow	100	-	-	-	-	100 (NR)

Abbreviations: DMT, disease-modifying therapy; FEES, fibreoptic endoscopic evaluation of swallowing; IQR, interquartile range; m, months; NBS, newborn screening; NR, not reported; Nu, nusinersen; OA, onasemnogene abeparvovec; PAS, penetration–aspiration scale; POS, prospective observational study; ROS, retrospective observational study; SD, standard deviation; SMA, spinal muscular atrophy; SMN, survival of motor neuron; VFSS, videofluoroscopic swallowing studies; W, weeks

^a^ Values correspond to percentages of patients (*N*), unless stated otherwise. Timepoint refers to time on treatment, unless specified

^b^ No timepoint specified for resolution of signs and symptoms of aspiration

^c^ Nine children received onasemnogene abeparvovec; five of these nine had already received nusinersen. One child received risdiplam

^d^ Patients with Type 1 SMA received nusinersen before receiving onasemnogene abeparvovec. Patients with Type 2 SMA received onasemnogene abeparvovec

Clinical trials are in italics

#### Presymptomatic SMA

The only investigation in presymptomatic SMA was Chiang et al. [32], and was limited to outcomes in a single patient.

#### Symptomatic SMA

Two of the three studies examining swallowing outcomes in patients treated after symptom onset were in children with Type 1 SMA. High rates of penetration or aspiration were observed after treatment: specifically, van der Heul et al. reported aspiration amongst 80% of children treated with nusinersen (timepoint unspecified) [4], and Zang et al. reported penetration or aspiration and pharyngeal residue in all but one (*n* = 9/10) patient following 6 months of treatment with varying DMTs [29]. These high rates of impaired swallowing biomechanics are in contrast with the results from AlNaimi et al. who reported ‘normal’ swallowing in three patients with Type 1 or Type 2 SMA after treatment with onasemnogene abeparvovec [31]. Biomechanical correlates constituting normal integrity were not reported [31].

Significant improvements in maximal mouth opening were reported in children with SMA after 3 months of treatment with onasemnogene abeparvovec in as single study [33].

### Functional swallowing outcomes

Functional swallowing outcomes were assessed using a variety of metrics. Oral intake status was the most common functional swallow outcome used (85%, 61/72 studies; Table 3). Other studies (19%,14/72; Table 4) reported results from a battery of standard patient-/caregiver-reported swallowing assessments, including Parent Assessment of Swallowing Ability (PASA), the Revised Amyotrophic Lateral Sclerosis Functional Rating Scale, Oral and Swallowing Abilities Tool (OrSAT), Syndey Swallow Questionnaire, Egen Klassifikation Scale Version 2, the Murray secretion scale, Jaw Functional Limitation Scale, and Individualised Neuromuscular Quality of Life questionnaire.

**Table 3:** Oral feeding status

DMT	Study (reference)	Study type	*N*	SMA type	*SMN2***copy number**	Age at onset Median (range) Mean [range/SD]	Age at treatment initiation/enrolment Median (range/IQR) Mean [range/SD]	Outcome assessed	**At timepoint of interest (%)**^a^					
									Baseline	6 months	12 months	18 months	24 months	Other (timepoint)
**Studies in presymptomatic SMA**														
Nusinersen	*NURTURE Sansone 2021* [35]	*SA*	*15*	*Presymptomatic*	*2*	*NR*	≤*6w*	*Full oral nutrition*	*-*	*-*	*-*	*-*	*84* *(Day 778)*	*-*
			*10*		*3*	*NR*	≤*6w*	*Not tube fed*	*-*	*-*	*-*	*-*	*100* *(Day 778)*	*-*
	*NURTURE* *Crawford 2023* [36]	*SA*	*25*	*Presymptomatic*	*2, 3*	*NR*	*22d* *(3–42)*	*PEG*	*-*	*4* *(at 5.9m)*	*-*	*-*	*8* *(at 19.4m and 22.5m)*	*8 (at 41.9m and 50.1m)*
Onasemnogene abeparvovec	*SPR1NT* *Strauss 2022* [21]	*SA*	*14*	*Presymptomatic*	*2*	*8.0d (1–14)*^*b*^ *7.2d* *[SD: 4.8]*	*21d* *(8–34)*	*Full oral nutrition*	*100*	*-*	*-*	*100*	*-*	*-*
	*SPR1NT* *Strauss 2020* [34]		*30*		*2–4*	*NR*	*2 SMN2 copies* *20.6d* *[8.0–34.0]* *3 SMN2 copies* *28.7d* *[9.0–43.0]*	*Full oral nutrition*	*-*	*-*	*-*	*-*	*-*	*100* *(CCOD: 31 Dec 2019)*
	*SPR1NT* *Post-hoc analysis* *Shell 2023* [22]	*SA*	*14*	*Presymptomatic*	*2*	*8.0d (1–14)* *7.2d* *[SD: 4.8]*^*b*^	*21d (8–34)* *20.6d* *[SD: 7.9]*	*Fully oral* *nutrition*	*100*	*-*	*-*	*100*	*-*	*-*
			*15*		*3*	*8.0d (2–26)*^*b*^ *9.9d* *[SD: 7.7]*	*32.0d* *(9–43)* *28.7d [SD:11.7]*		*100*	*-*	*-*	*-*	*100*	*-*
Risdiplam	*RAINBOWFISH Finkel 2022* [23]	*SA*	*7*	*Presymptomatic*	*2, 3, 4*	*NR*	*26.5d* *(16–40)* *(n = 18)*	*Able to feed orally*	*100*	*-*	*100*	*-*	*-*	*-*
**Studies in a Type 1 SMA population**														
Nusinersen	De Lucia 2020 [24]	ROS	23	Type 1	2, 3	NR	NR	Gastrostomy/NG tube	18.0	-	-	56.0	-	-
	Sansone 2020 [37]	EAP	118	Type 1	1–4	NR	42.8m (IQR:11.0–102.8)	NG tube	10.2	-	-	-	-	5.7 (T300) *N* = 105
								PEG	54.2	-	-	-	-	65.7 (T300) *N* = 105
	Lavie 2020 [38]	POS	20	Type 1	2, 3	-	13.5m (1–184) IQR: 4–56	Gastrostomy/ NG tube	65.0	-	-	-	95.0	-
	van der Heul 2020 [4]	POS	5	Type 1	2	38d (21–90)	63d (3–218)	Tube feeding	-	-	-	-	-	100 (median start at 382d)
	Bianchi 2021 [39]	POS	10	Type 1	2, 3	2.5m (0–5)	9.5m (2–28) [SD: 10.02]	Required tube feeding	50.0	60.0^c^	-	-	-	-
	Chen 2021 [40]	ROS	9	Type 1	2, 3	3m (1–5)	10.6m (2.7–178.6)	Gastrostomy	22.0	-	-	-	-	66.7 (30.1m)
	Mendonca 2021 [41]	POS	21	Type 1	2, 3	2.7m [SD: 1.5]	NR	Feeding orally	4.8	4.8^d^	-	-	-	-
								Oral + gastrostomy	9.5	-	-	-	-	9.5^d^
								Gastrostomy	85.7	-	-	-	-	85.7^d^
	Modrzejewska 2021 [42]	POS	26	Type 1	2, 3, 4	2m (0–6) 2m [SD: 1.72]	23m (3–165) 36.57m [SD: 39.25]	PEG/g astrostomy	57.7	-	-	-	-	50.0 (18–26m)
	Pane 2021 [43]	EAP	68	Type 1	1–4	NR	3.96 [0.20–15.92] [SD: 3.90]	Orally fed	47.1	-	-	-	35.3	-
								Tube fed	52.9	-	-	-	64.7	-
	Ergenekon 2022 [44]	ROS	52	Type 1	2, 3	2.2m [SD: 1.8]	11.3m (4.0–34.8)	Able to feed orally	42.3	32.6 *N* = 46	-	-	-	-
								Gastrostomy	21.2	34.8 *N* = 46	-	-	-	-
								NG feeding	36.5	32.6 *N* = 46	-	-	-	-
	Menard 2022 [45]	ROS	17	Type 1	2, 3	2m (1–6)	4m (2–29)	NG tube	-	-	-	-	-	52.9 (mean: 10m; IQR 4–15)
								Gastric feeding tube	-	-	-	-	-	64.7 (mean: 16m; IQR 14 −23.5)
	Weststrate 2022 [15]	ROS	24	Type 1	2, 3	NR	11m [1m–7y 6m]	p-FOIS median score	3	1	2	2	-	-
								Tube feeding	58.3	-	83.3	-	83.3	
			3	Type 1a	2, 3	NR	2y 2m (1m–4y 3m)	p-FOIS median score	1	1	1	1	-	-
								Tube feeding	100	-	100	-	100	-
			9	Type 1b	2	NR	4m (2m–1y 4m)	p-FOIS median score	3	1	2	2	-	-
								Tube feeding	55.6	-	100	-	100	-
			12	Type 1c	2, 3	NR	1y 7m (8m–7y 6m)	p-FOIS median score	5	2	3	3	-	-
								Tube feeding	50.0	-	66.7	-	66.7	-
	Pane 2023b [46]	EAP	48	Type 1	1–3	NR	3.3y [7d–12y] [SD: 3.6]	Oral feeding	52.1	-	-	-	-	41.7 (48 m)
								Gastrostomy or NG tube	47.9	-	-	-	-	58.3 (48 m)
	Pechmann 2023b [47] SMARTcare registry	POS	88	Type 1^e^ ≤2 years of age at start of treatment	1–≥4	2m (0–10) 2.8m [SD: 2.5]	7m (0–24) 8.4m [SD: 6.0]	No tube feeding	70.5	-	-	-	-	-
								Tube feeding supplementary	19.3	-	-	-	-	-
								Tube feeding exclusively	10.2	-	-	-	-	-
			55	Type 1^e^ >2 years of age at start of treatment	1–≥4	5m (0–56) 6.2m [SD: 7.8]	68m (24–207) 89.8m [SD: 58.4]	No tube feeding	61.8	-	-	-	-	-
								Tube feeding supplementary	25.5	-	-	-	-	-
								Tube feeding exclusively	12.7	-	-	-	-	-
								Probability of the need for tube feeding	43.6	-	-	-	-	51.4 (38m)
	Shin 2023 [48]	ROS	7	Type 1	2, 3	NR (3.0–4.4m) 3.7m [SD: 1.4]	NR (6.4–9.4y) 7.3y [SD: 4.0]	Orogastric	14.3	-	-	-	-	14.3 (46.2m)
								Gastrostomy	85.7	-	-	-	-	85.7 (46.2m)
	Xiao 2023 [49]	ROS	11	Type 1	2	1.5m (0.5–4.0)	5.4m (2.0–108.7)	Enteral feeding	54.5	-	-	-	-	NR
Onasemnogene abeparvovec	*START* *Mendell 2017* [50]	*DC/DE*	*12*	*Type 1*	*2*	*Cohort 2* *1.4m* *[0–3.0]*	*Cohort 2* *3.4m* *[0.9–7.9]*	*Required enteral feeding*	*41.7*	*-*	*-*	*-*	*-*	*-*
								*Oral nutrition*	*64*	*-*	*-*	*-*	*-*	*-*
	*Alecu 2021* *START LTFU* [51]	*POS*	*13*	*Type 1*	*2*	*NR*	*0.3y* *(0.1–0.6)* *0.3y* *(SD: 0.2)*	*Did not require feeding support*	*-*	*-*	*-*	*-*	*-*	*60* *(CCOD: Dec 2019)*
								*Fed orally*	*-*	*-*	*-*	*-*	*-*	*100* *(CCOD: Dec 2019)*
	*START* *McGrattan 2023* [25]	*SA* *Post-hoc analysis*	*11*	*Type 1*	*2*	*NR*	*NR*	*Full oral nutrition*	*63.6*	*-*	*-*	*-*	*54.5 (at 24m of age)*	*-*
	*STR1VE-EU Mercuri 2021* [26]	*SA*	*33*	*Type 1*	*2*	*1.5m* *(0.0–4.0)* *1.6m* *[SD: 0.9]*	*4.1m* *(1.8–6.0)* *4.1m* *[SD: 1.3]*	*Required feeding support*	*27.3*	*-*	*-*	*15.2 (at 18m of age)*	*-*	*-*
	*STR1VE-EU* *McGrattan 2023* [25]	*SA* *Post-hoc analysis*	*32*	*Type 1*	*2*	*NR*	*NR*	*Full oral nutrition*	*81.2*	*-*	*-*	*75.0*	*-*	*-*
	*STR1VE-US* *Day 2021* [27]	*SA*	*22*	*Type 1*	*2*	*1.8m* *(1.0–3.0)* *1.9m* *[SD: 1.2]*	*3.5m* *(2.7–5.3)* *3.7m* *[SD: 1.6]*	*Able to swallow thin liquid*	*100*	*-*	*-*	*54.5 (at 18m of age)*	*-*	*-*
								*Did not require any non-oral feeding support*	*100*	*-*	*-*	*-*	*-*	*-*
								*Fed exclusively by mouth*	*-*	*-*	*-*	*86.4 (at 18m of age)*	*-*	*-*
								*Did not require feeding support at any point during study*	*-*	*-*	*-*	*68.2 (at 18m of age)*	*-*	*-*
	*STR1VE-US* *McGrattan 2023* [25]	*SA* *Post-hoc analysis*	*22*	*Type 1*	*2*	*NR*	*NR*	*Full oral nutrition*	*100*	*-*	*-*	*86.4 (at 18m of age)*	*-*	*-*
	*START STR1VE-EU* *STR1VE-US* *McGrattan 2023* [25]	*SA* *Post-hoc analysis*	*65*	*Type 1*	*2*	*NR*	*NR*	*Full oral nutrition*	*84.6*	*-*	*-*	*-*	*-*	*75.4* *(aged 18m and 24m)*
	Bitetti 2022 [52]	POS	9	Type 1	2	2.7m [0–5] [SD: 1.9]	NR	PEG tube	22.2	-	-	-	-	22.2 (3m)
	Gowda 2023 [53]	ROS	15	Type 1	NR	NR	NR (2–38m)	Fully orally fed	40.0	-	-	-	-	40.0 (8–15m)
								Fully tube fed	40.0	-	-	-	-	40.0 (8–15m)
								Mixed oral and tube feeding	13.3	-	-	-	-	6.7 (8–15m)
Risdiplam	*FIREFISH* *Part 1* [54]	*DC/* *DE*	*21*	*Type 1*	*2*	*2.0m* *(0.9–3.0)*	*6.7m* *(3.3–6.9)*	*Able to feed orally*	*-*	*-*	*85.7*	*-*	*-*	*-*
								*Combination of oral and tube feeding*	*-*	*-*	*14.3*	*-*	*-*	*-*
	*FIREFISH* *Part 2* [28, 55]	*SA*	*41*	*Type 1*	*2*	*1.5m* *(1.0–2.0)*	*5.3m* *(4.2–6.8)*	*Able to feed orally*	*85.4*	*-*	*82.9*	*-*	*85.4*	*-*
								*Fed exclusively via feeding tube*	*9.8*	*-*	*-*	*-*	*7.3*	*-*
								*Fed via combination of oral and feeding tube*	*4.9*	*-*	*14.6*	*-*	*14.6*	*-*
								*Fed exclusively orally*	*80.5*	*-*	*68.3*	*-*	*70.7*	*-*
	FIREFISH Part 1 + Part 2 pivotal dose cohort [55]	SA	58	Type 1	2	-	-	*Able to feed orally*	*-*	*-*	*84*	*-*	*83*	*-*
Risdiplam switch to onasemnogene abeparvovec	Quelch 2023 [56]	ROS	5	Type 1	NR	5.8m (1–12)	26m (7–87)	Exclusively oral feeding	40.0	-	-	-	-	-
								Exclusively NG feeding	40.0	-	-	-	-	-
								Mixed NG/oral feeding	20.0	-	-	-	-	-
Onasemnogene abeparvovec (*n* = 4); nusinersen switching to onasemnogene abeparvovec (*n* = 5); risdiplam (*n* = 1)	Zang 2023 [29]	POS	10	Type 1	2	2.0m (0.0–7.0)	3.8m (0.7–8.9)	NdSSS median score	4.5	3.0	-	-	-	-
**Studies with a Type 2/3 SMA population**														
Nusinersen	Ambawatte 2022 [30]	ROS	9	Type 2	3,4	NR	NR	Gastrostomy	11.1	-	-	-	-	-
	Vetlesen 2024 [57]	POS	20	Type 2	3, 4	NR	7.0y (IQR: 2.0–12.9)	Gastrostomy	5.0	-	-	-	-	5.0 (3y)
			20	Type 3	2, 3, 4	NR	8.4y (IQR: 4.3–12.2)	Gastrostomy	0	-	-	-	-	0 (3y)
**Studies with a mixed SMA type population**														
Nusinersen	Aragon-Gawinska 2018 [94]	POS	15	NR	2	3m (1.5–5)	19.8m (8.3–42.0)	No nutritional support	73	60	-	-	-	60 (M2)
								Tube feeding	27	40	-	-	-	40 (M2)
			17	NR	3	4m (2–6)	27.7 (8.8–113.1)	No nutritional support	71	71	-	-	-	71 (M2)
								Tube feeding	29	29	-	-	-	29 (M2)
			33	NR	2, 3	4m (1.5–6)	21.3m (8.3–113.1)	No nutritional support	73	64	-	-	-	67 (M2)
								Tube feeding	27	36	-	-	-	33 (M2)
	Audic 2020 [63]	ROS	30	Type 1/2 (age <2y)	2–4	NR	(3m–17y)	NG tube/gastrostomy	10.0	-	16.7	-	-	-
			47	Type 1/2 (age 2–5y)				NG tube/gastrostomy	12.8	-	12.8	-	-	-
			46	Type 1/2 (age 6–17y)				NG tube/gastrostomy	10.9	-	10.9	-	-	-
	Kim 2020 [61]	ROS	4	Type 1	2	(7d–5m)	NR	Gastrostomy	50.0	-	-	-	-	50.0 (median F/U 6.1m)
								Oral feeding without difficulty	50.0	-	-	-	-	50.0 (median F/U 6.1m)
			3	Type 2	3	10.67m (8–15)	NR	Gastrostomy	0	-	-	-	-	0 (median F/U 6.1m)
								Oral feeding without difficulty	100	-	-	-	-	100 (median F/U 6.1m)
	Osredkar 2021 [64]	POS	61	Types 1–3	2–4	NR	8.6y (0.2–18.8)	Gastrostomy	13.1	-	-	-	-	14.8 (14m)
	Barisic 2022 [95]	ROS	18	Type 1	NR	NR	3m–3.5y	Required feeding support	16.7	-	-	-	-	16.7 (36m)
	Calvo-Medina 2022 [96]	ROS	20	Type 1/2	2–4	7m (IQR: 4–12)	4 y (IQR: 19m–11)	NG tube/gastrostomy	-	-	-	-	-	20.0 (34m)
	Hepkaya 2022 [65]	ROS	43	Types 1–3	<3 copies ≥3 copies	27.8 [SD: 39.1]^b^	60.8 [SD: 69.5]	NG tube	9.3	-	-	-	-	18.6 (T1)^f^ 14.3 (T2)^f^
								Gastrostomy	2.3	-	-	-	-	4.7 (T1)^f^ 9.3 (T2)^f^
								Oral feeding	88.4	-	-	-	-	76.7 (T1)^f^ 76.7 (T2)^f^
	Pechmann 2022a [59]	POS	256	Types 2/3	1–≥4	NR	NR	Tube feeding	5.5	-	-	-	-	7.4 (F/*U* ≤38m; CCOD Nov 2021)
	Pechmann 2023a [62] SMARTcare registry	POS	114	Type 3	2–≥4	40.8 [99% CI 30.4–51.5]	103.3 [99% CI 89–117.6]	Tube feeding	0	-	-	-	-	0 (38m)^g^
	Tscherter 2022 [97]	POS	6	Type 1 (aged 0–18m)	2, 3	NR	0.2y (0.1–0.4)	Tube feeding	0	-	-	-	-	66.7 (median F/U 25.5m)
	Belancic 2023 [58]	ROS	52	Type 1–3	1–4	NR	13.4y (0.1–51.1) 16.4y [SD: 13.2]	PEG	7.7	-	-	-	-	9.6 (completed the first 6 doses)
								NG tube	19.2	-	-	-	-	17.3 (completed the first 6 doses)
			18	Type 1	1–3	NR	5.2y (0.1–20.8) 6.3y [SD: 6.3]	PEG	22.2	-	-	-	-	27.8 (completed the first 6 doses)
								NG tube	44.4	-	-	-	-	38.9 (completed the first 6 doses)
			6	Type 2	1–3	NR	10.2y (1.5–16.7) 8.8y [SD: 6.0]	PEG	0	-	-	-	-	0 (completed the first 6 doses)
								NG tube	33.3	-	-	-	-	33.3 (completed the first 6 doses)
			28	Type 3	2–4	NR	24.8y (4.2–51.1) 24.5y [SD: 12.3]	PEG	0	-	-	-	-	0 (completed the first 6 doses)
								NG tube	0	-	-	-	-	0 (completed the first 6 doses)
	Dabbous 2023 [70]	ROS	19	Types 1–3	NR	NR	NR	Improved/ maintained in eating function	-	-	-	-	-	58.8 (*n* = 17)
	Johnson 2023 [67]	ROS	27	Unclear	NR	NR	NR	Gastrostomy	-	70.0 (0–6m)	52.0 (6–12m)	-	26.0 (12–24m)	-
	Öz Yıldız 2023 [98]	ROS	18	Type 1	NR	NR	6.5m (1–123)	PEG	-	-	-	-	-	11.1 (37.7m)
								NG tube	-	-	-	-	-	5.6 (37.7m)
	Toro 2023 [99]	ROS	62	Types 1/2	NR	NR	13.1 [SD: 7.1] [IQR: 7.0–21.0]	Nutritional support	-	-	-	-	-	74.2 (10m)
								NG tube	-	-	-	-	-	72.6 (10m)
	Audic 2024 [66]	ROS	57	Types 1/2	2, 3	NR	16m (2–34)	NG tube	3.5	-	-	-	-	0 (36m)
								Gastrostomy	7.0	-	-	-	-	24.6 (36m)
Onasemnogene abeparvovec	*LT-002* *Darras 2023* [100]	*SA*	*25*	*Presymptomatic* *IV*	*NR*	*NR*	*NR*	Nutritional support	*-*	*-*	*-*	*-*	*-*	*0 (CCOD May 2022)*
			*38*	*Symptomatic* *IV*	*NR*	*NR*	*NR*	Nutritional support	*-*	*-*	*-*	*-*	*-*	*20.0 (CCOD May 2022)*
			*63*	*Presymptomatic/symptomatic* *IV total*	*NR*	*NR*	*NR*	Able to feed orally	*-*	*-*	*-*	*-*	*-*	*95 (CCOD May 2022)*
			*18*	*IT*	*NR*	*NR*	*NR*	Nutritional support	*-*	*-*	*-*	*-*	*-*	*0 (CCOD May 2022)*
								Able to feed orally	*-*	*-*	*-*	*-*	*-*	*100 (CCOD May 2022)*
	Pane 2023a [101]	POS	46	Presymptomatic + Type 1	2/3	NR	NR	Did not need nutritional support	78.3	-	78.3	-	-	-
								Required tube feeding	21.7	-	21.7	-	-	-
	RESTORE Finkel 2023 [102]		19	NR	≥4	NR^h^	3 (1–11)	Nutritional support	**-**	**-**	**-**	**-**	**-**	0 (CCOD Dec 2022)
	RESTORE Servais 2024 [103]	POS	168	Types 1–3	Unknown,1, 2, 3, ≥4	NR	3 (1–10) 6.38 [SD: 8.29]	Fed exclusively by mouth	**-**	-	96.6	**-**	**-**	-
	Wang 2023 [68]	ROS	4	Type 1/2	NR	NR	NR	Weaned off NG tube to oral feeding	**-**	**-**	**-**	**-**	**-**	25.0 (NR)
	Waldrop 2020 [69]	ROS	21	Presymptomatic + symptomatic SMA	2–4	NR	(1–23m)	Feeding orally (exclusively and partially)	76.2	**-**	**-**	**-**	**-**	80.9 (NR)
	Dabbous 2023 [70]	ROS	8	Types 1–3	NR	NR	NR	Subjective improvement/ maintenance in eating function	**-**	**-**	**-**	**-**	**-**	83.3 (NR) *N* = 6
	Stettner 2022 [104]	POS	6	Type 1	NR	NR	5m [2w–17m]	Required nutritional support	-	-	-	-	-	67 (270d)
	Chiang 2023 [32]	ROS	11	Unclear	2, 3	NR	3.6w (IQR: 3.3–21.3)	Enteral feeding	0	-	-	-	-	0 (NR)
	Martins 2023 [71]	POS	25	Type 1/2	NR	NR	NR	Stabilisation or improvement in oral function	-	-	100	-	-	-
								Gastrostomy	-	-	4	-	-	-
	Toro 2023 [99]	ROS	12	Types 1/2	NR	NR	14.2 [SD: 6.7] (IQR: 8.8–20.5)	Nutritional support	-	-	-	-	-	50.0 (9.8m)
								Gastrostomy	-	-	-	-	-	41.7 (9.8m)
	Waldrop 2024 [72]	ROS	19	Unclear	2–4	NR	(7–729d)	Unable to eat orally	52.6	-	-	-	-	-
								Improved in oral feeding status	-	-	-	-	-	36.8 (33.3m)
			46					Decline in oral feeding status	-	-	-	-	-	0 (33.3m)
Nusinersen, onasemnogene abeparvovec	AlNaimi 2022 [31]	ROS	9	Type 1	2	2m (0.3–6)^b^	12m (4–23)	Enteral feeding	55.6	-	-	-	-	55.6 (F/U 6–24m)
Nusinersen, onasemnogene abeparvovec, risdiplam	Segovia 2021 [105]	ROS	21	Type 1	NR	NR	4y [0–9]^i^	Tube feeding	41.1 (NR)	-	-	-	-	-
			62	Type 2	NR	NR	11y (2–37)^i^	Tube feeding	2.5 (NR)	-	-	-	-	-
Nusinersen switched to onasemnogene abeparvovec	Dabbous 2023 [70]	ROS	15	Types 1–3	NR	NR	NR	Subjective improvement/ maintenance in eating function	-	-	-	-	-	50.0 (NR) *N* = 14
Nusinersen monotherapy, onasemnogene abeparvovec monotherapy, nusinersen followed by risdiplam	Angeli 2023 [106]	ROS	11	Presymptomatic/Type 1/2/3	NR	NR	61.7m (SD: 66.5)	NG tube or gastrostomy	-	-	-	-	-	0 (43m)
Onasemnogene abeparvovec Nusinersen switch to onasemnogene abeparvovec	Latzer 2023 [60]	ROS	6	Type 1a	2	1.62m [0.1–2.5]	3.69m [0.75–6]	Oral feeding	66.7	-	-	33.3	-	-
								Enteral feeding	33.3	-	-	66.7	-	-
			14	Type lb	2, 3	1.96m [0.2–4.0]	10.51m [0.3–24]	Oral feeding	92.9	-	-	71.4	-	-
								Partially oral and enteral feeding	0	-	-	14.3	-	-
								Enteral feeding	7.1	-	-	14.3	-	-
			3	Type 1c	3, 4	4.4m [4.1–5.0]	9.9m [4.7–18]	Oral feeding	100	-	-	100	-	-
			2	Type 2	3	10.55m [7.1–14]	20m [19–21]	Oral feeding	100	-	-	100	-	-
			25	Types 1/2	2–4	-	6.1m (3.3–17.0)	Enteral feeding	12	-	-	24	-	-
								Oral feeding	88	-	-	68	-	-
								Partially oral and enteral feeding	0	-	-	8	-	-
Risdiplam (*n* = 45); nusinersen (*n* = 20); onasemnogene abeparvovec (*n* = 4)	Vuillerot 2023 [107]	**ROS**	11	Type 1	NR	NR	NR	Fed exclusively by mouth	-	-	-	-	-	81.8 (1–8y^j^)

Abbreviations: CCOD, clinical cut-off date; CI, confidence interval; d, days; DMT, disease-modifying therapy; CCOD, clinical cut-off date; DC, dose comparison; DE, dose escalation; EAP, expanded access programme; F/U, follow-up; IQR, interquartile range; IT, intrathecal; IV, intravenous; LTFU, long-term follow-up; m, months; NdSSS, Neuromuscular Disease Swallowing Status Scale; NG, nasogastric; NR, not reported; PEG, percutaneous endoscopic gastrostomy; p-FOIS; Paediatric Functional Oral Intake Scale Score; POS, prospective observational study; ROS, retrospective observational study; SA, single arm; SD, standard deviation; SMA, spinal muscular atrophy; SMN, survival of motor neuron; T1, timepoint 1, T2, timepoint 2; T300, 300 days post-infusion; w, weeks; y, years

^a^ Values are percentages of patients (*N*), unless otherwise stated. Timepoint refers to time on treatment, unless specified

^b^ Age at diagnosis

^c^ One patient who did not require nutritional support at baseline required an NG tube at Day 180 and one patient who required an orogastric tube at baseline had received a PEG by Day 180

^d^ Six infants were followed up for 6 months, five for 12 months, seven for 16 months, and three for 24 months. No infant changed their feeding status over the duration of F/U. One infant with gastrostomy died within 6 months of F/U

^e^ All patients under treatment with nusinersen who never had the ability to sit independently before the start of treatment were identified for data analysis

^f^ T1 was performed after the nusinersen loading doses were administered – Day 64 for Type 1 SMA and Day 274 for Types 2/3 SMA. T2 was performed 4 months after the first maintenance dose of nusinersen was given: ~Day 187 for Type 1 SMA and ~Day 643 for Types 2/3 SMA

^g^ One paediatric patient (0.9%) required tube feeding for a period of 6 months during the study

^h^ All but one patient received presymptomatic treatment

^i^ Age at baseline

^j^ A retrospective study of patients followed in a neuromuscular disease reference centre between 2012 and 2022

Clinical trials are in italics

**Table 4:** Patient-reported swallowing and feeding outcomes

DMT	Study (reference)	Study type		*N*	SMA type	SMN2 copy number	Age at onset Median (range) Mean [range/SD]	Age at treatment initiation/enrolment Median (range/IQR) Mean [range/SD]	Outcome assessed	At timepoint of interest (%)^a^					
										Baseline	6 months	12 months	18 months	24 months	Other (timepoint)
**Studies in presymptomatic SMA**															
Nusinersen	NURTURE Day 778: Sansone 2021 [34] 5y: Crawford 2023 [35]	SA		25	Presymptomatic	2/3	*NR*	22.0d (3–42)	PASA: able to swallow	-	-	-	-	92 (Day 778)	92 (5y)
									PASA: not being tube fed	-	-	-	-	84 (Day 778)	80 (5y)
									PASA: never gagged/choked on liquid food	-	-	-	-	91 (Day 778)	-
									PASA: never gagged/choked on solid food	-	-	-	-	87 (Day 778)	-
									PASA: no swallowing concerns over choking^b^	-	-	-	-	88 (Day 778)	76 (5y)
									PASA: no aspiration concerns whilst eating^b^	-	-	-	-	96 (Day 778)	80 (5y)
				15		2	*NR*	≤6w	PASA: not being tube fed	-	-	-	-	73 (Day 778)	67 (5y)
				10		3	*NR*	≤6w	PASA: not being tube fed					100 (Day 778)	100 (5y)
**Studies in a Type 1 SMA population**															
Nusinersen	Berti 2022 [73]	POS		18	Type 1 – all	NR	NR	6.5m [3w–15m]	Mean %-change (SD) in OrSAT score	-	-	-	−5.45% (28.69)	-	-
				8	Type 1 – patients who never had PEG/tracheostomy				Mean %-change (SD) in OrSAT score	-	-	-	+1.79% (20.02)	-	-
				2	Type 1 – patients who required tube feeding after treatment started				Mean %-change (SD) in OrSAT score	-	-	-	−67.86% (33.67)	-	-
				4	Type 1 – patients who required PEG, but no tracheostomy				Mean %-change (SD) in OrSAT score	-	-	-	+5.83% (20.2)	-	-
				4	Type 1 – patients who required PEG and tracheostomy				Mean %-change (SD) in OrSAT score	-	-	-	0.00%	-	-
	Cho et al. 2023 [108]	ROS		21	Type 1	1–4	≤3y	2.3y [SD: 4.6]	Improvement in swallowing and speech	-	-	20	-	-	-
Nusinersen, onasemnogene abeparvovec, risdiplam	Zang 2023 [29]	POS		10	Type 1	2	2.0m (0.0–7.0)	3.8m (0.7–8.9)	Murray secretion scale: median score	2	1.5	-	-	-	-
									OrSAT median score	3.5	3	-	-	-	-
**Studies with a Type 2/3 SMA population**															
Nusinersen	SHINE–CHERISH [74]	SA	119		Type 2/3	*NR*	*NR*	*NR*	PASA – tube feeding	2.5	-	-	-	-	1.7 (3.7y)
	Brakemeier 2021 [109]	POS	22		Types 2/3	3 to ≥6	NR	38.5y [SD: 14.2] (20–72)	SSQ bulbar sub score – mean (SD)	23.6 (18.9) *n* = 18	24.1 (19.3) *n* = 15	-	-	-	20.0 (17.9) (*n* = 13, 14m)
									ALSFRS-R bulbar sub score – mean (SD)	10.5 (1.2) *n* = 16	10.5 (1.3) *n* = 20	-	-	-	10.9 (1.0) (*n* = 18, 14m)
	Cho et al. 2023 [108]	ROS	103		Type 2	1–4	≤3 years	15.4y [SD: 10.0]	Improvement in swallowing/speech	-	-	5	-	-	-
	Vetlesen 2024 [57]	POS	40		Types 2/3	2–4	NR	7.2y (2.4–12.7)	EK2 – feeding difficulty^c^	-	-	-	-	-	65 (3y)
									EK2 – difficulties with food textures^c^	-	-	-	-	-	37.5 (3y)
									EK2 – prolonged mealtimes^c^	-	-	-	-	-	42.5 (3y)
									EK2 – swallowing difficulties^c^	-	-	-	-	-	22.5 (3y)
			20		Type 2	3/4	NR	7.0y (2.0–12.9)	Feeding difficulty^c^	-	-	-	-	-	85 (3y)
									EK2 – difficulties with food textures^c^	-	-	-	-	-	50 (3y)
									EK2 – prolonged mealtimes^c^	-	-	-	-	-	65 (3y)
									EK2 – swallowing difficulties^c^	-	-	-	-	-	35% (3y)
			20		Type 3	2–4	NR	8.4y (4.3–12.2)	EK2 – feeding difficulty^c^	-	-	-	-	-	45 (3y)
									EK2 – difficulties with food textures^c^	-	-	-	-	-	25 (3y)
									EK2 – prolonged mealtimes^c^	-	-	-	-	-	20 (3y)
									EK2 – swallowing difficulties^c^	-	-	-	-	-	10 (3y)
	DEVOTE (Part A) Finkel 2023 [110]	SA	6		Type 3	3/4	19.5m (8–36)	9.4y (6.1–12.6)	PASA score: not able to eat as much as would like	3.5	-	-	3.7 (302d)	-	3.7 (64d) 3.7 (269d)
									PASA score: had to suction excess saliva or drool	4	-	-	4 (302d)	-	4 (64d) 4 (269d)
									PASA score: not able to eat food variety they like	3.8	-	-	3.7 (302d)	-	3.7 (64d) 3.7 (269d)
									PASA score: had difficulty feeding themselves	3.3	-	-	3.5 (302d)	-	3.5 (64d) 3.5 (269d)
									PASA score: cough/clear throat - swallow liquid food	4	-	-	4 (302d)	-	4 (64d) 4 (269d)
									PASA: gagged or choked on liquid food	4	-	-	4 (302d)	-	4 (64d) 4 (269d)
									PASA: refused liquid food	4	-	-	4 (302d)	-	4 (64d) 4 (269d)
									PASA: retching/vomiting when drinking liquids	4	-	-	4 (302d)	-	4 (64d) 4 (269d)
									PASA: taken >30 minutes to drink liquids	4	-	-	4 (302d)	-	4 (64d) 4 (269d)
									PASA: difficulty swallowing soft foods	4	-	-	4 (302d)	-	4 (64d) 4 (269d)
									PASA: difficulty swallowing solid foods	3.8	-	-	3.8 (302d)	-	3.8 (64d) 3.8 (269d)
									PASA: experienced or shown pain when eating	4	-	-	4 (302d)	-	4 (64d) 4 (269d)
									PASA: gagged or choked on solid food	3.8	-	-	4 (302d)	-	3.8 (64d) 3.8 (269d)
									PASA: has taken >30 minutes to eat solids	3.0	-	-	3.3 (302d)	-	3.0 (64d) 3.2 (269d)
									PASA: refused solid foods	3.8	-	-	4 (302d)	-	3.8 (64d) 3.7 (269d)
									PASA: retching/vomiting when eating solids	3.7	-	-	3.8 (302d)	-	3.7 (64d) 3.8 (269d)
									PASA: had difficulty swallowing pills	3.7	-	-	3.8 (302d)	-	4 (64d) 4 (269d)
									PASA: required food to be cut up	2.8	-	-	3.5 (302d)	-	3.5 (64d) 3.5 (269d)
									PASA score: been tube fed	4	-	-	4 (302d)	-	4 (64d) 4 (269d)
									PASA score: concern unable to eat as much as like	1.3	-	-	2.5 (302d)	-	2.6 (64d) 1.6 (269d)
									PASA score: concern unable to eat variety as like	1.5	-	-	2.8 (302d)	-	2.5 (64d) 1.8 (269d)
									PASA score: concerned about my child’s weight	0.8	-	-	2.0 (302d)	-	2.0 (64d) 1.5 (269d)
									PASA score: concerned about swallowing ability	2.0	-	-	2.8 (302d)	-	2.8 (64d) 1.6 (269d)
									PASA score: concerned about variety of foods eaten	1.3	-	-	2.0 (302d)	-	2.5 (64d) 1.8 (269d)
									PASA score: concerned about aspirating food	2.0	-	-	3.0 (302d)	-	2.8 (64d) 2.0 (269d)
									PASA: concerned about choking when eating	1.8	-	-	3.0 (302d)	-	2.8 (64d) 2.0 (269d)
									PASA score: concerned about not getting goodness from diet	1.0	-	-	2.8 (302d)	-	2.1 (64d) 1.8 (269d)
Risdiplam	Zoppi 2022 [75]	POS	6		Types 2/3	NR	NR	40y (22–25)	Reported a subjective benefit with bulbar function	-	-	-	-	-	100 (FU: 6–21 m)
	Ñungo Garzόn 2023 [76]	EAP	6		Type 2	1, 3, 4	NR	NR	EK2 – bulbar function score	4.33 (mean) 4 (median)	-	3.5 (mean) 3 (median)	-	-	-
									ALSFRS-R –bulbar function score	7.83 (mean) 8.5 (median)	-	10.5 (mean) 10.5 (median)	-	-	-
	Brakemeier 2024 [77]	POS	25		Types 2/3	2/3	NR	34.3 [11.3] (19–58)	SSQ bulbar sub score – mean (SD)	19.5 (16.4) *n* = 24	-	15.6 (18.6) *n* = 19	-	-	17.4 (16.9) *n* = 23 (4m)
									SSQ bulbar sub score – improved	-	-	83	-	-	64 (4m)
									SSQ bulbar sub score – unchanged	-	-	0	-	-	9 (4m)
									SSQ bulbar sub score – declined	-	-	17	-	-	27 (4m)
									ALSFRS-R bulbar sub score mean (SD)	10.1 (1.3) *n* = 24	-	10.4 (1.5) *n *= 17	-	-	10.3 (1.7) *n* = 22 (4m)
									ALSFRS-R – improved	-	-	47			41 (4m)
									ALSFRS-R – unchanged	-	-	35			41 (4m)
									ALSFRS-R – declined	-	-	18			18 (4m)
	Sitas 2024 [78]	ROS	15		Type 2	2–4	2.0y (0.5–18)	30y (18–65)	JFLS – mouth opening limitations indicative of TMJ contracture	100	-	-	-	-	60 (2.5y)
									JFLS – swallowing difficulties	86.7	-	-	-	-	-
									JFLS –improvement in swallowing	-	-	-	-	-	20 (2.5y)
			16		Type 3				JFLS – swallowing difficulty	12.5	-	-	-	-	-
			31		Type 2/3				INQoL – swallowing improvement	-	-	-	-	-	26.7 (2.5y)
**Studies in a mixed SMA population**															
Nusinersen	Osredkar 2021 [64]	POS		61	Types 1–3	2–4	NR	Type 1 5.2y (0.2–14.7) Type 2 8.9y (0.8–18.8) Type 3 12.6y (1.9–18.6)	Improvement in aspirations	-	-	-	-	-	3.3 (14m)
									Improvement in feeding	-	-	-	-	-	26.2 (14m)
									Improvement in salivation	-	-	-	-	-	0 (14m)
Risdiplam	Gomez Garcia de la Banda 2022 [111]	ROS		15	Types 1–3	NR	NR	NR	Improvement in swallowing	-	-	-	-	-	6.67 (≥12m)
Nusinersen, onasemnogene abeparvovec Onasemnogene abeparvovec + nusinersen (combo therapy)	D’Silva 2022 [112]	POS		21	Unclear	2, 3	27d (9–329)^b^	11m (0.65–24)	OrSAT: normal oral and swallowing abilities^d^	66.7	-	-	-	-	71.4 (15m)
									OrSAT: moderate impairment: supplemental nutrition required (NG, nasojejunal, or gastrostomy tube)^d^	4.7	-	-	-	-	9.5 (15m)
									OrSAT: all nutrition and hydration via non-oral means^d^	28.5	-	-	-	-	19.0 (15 m)

Abbreviations: ALSFRS-R, Revised Amyotrophic Lateral Sclerosis Functional Rating Scale; d, days; DMT, disease-modifying therapy; EAP, expanded access programme; EK2, Egen Klassifikation Scale Version 2; INQoL, Individualised Neuromuscular Quality of Life questionnaire; IQR, interquartile range; JFLS, Jaw Functional Limitation Scale; m, months; NG, nasogastric; NR, not reported; OrSAT, Oral and Swallowing Abilities Tool; PASA, Parent Assessment of Swallowing Ability; PEG, percutaneous endoscopic gastrostomy; POS, prospective observational study; ROS, retrospective observational study; SA, single arm; SD, standard deviation; SMA, spinal muscular atrophy; SMN, survival of motor neuron; SSQ, Syndey Swallow Questionnaire; TMJ, temporomandibular joint; w, weeks; y, years

^a^ Values are percentages of patients (*N*), unless otherwise stated. Timepoint refers to time on treatment, unless specified

^b^ Parents disagreed/strongly disagreed with the PASA statements

^c^ Feeding difficulties are defined as scoring >0 in EK2 total score or within the individual EK2 function domains

^d^ The OrSAT was applied through retrospective analysis of patient records. No mention of patient report

Clinical trials are in italics

#### Oral intake status

##### Presymptomatic SMA

Results from six publications indicate that most patients, regardless of *SMN2* copy number and treatment type, were taking full oral nutrition at the last follow-up (range: 5.9–50.1 months). All studies investigating outcomes after onasemnogene abeparvovec and risdiplam reported that 100% of infants had full oral intake at the last timepoint (12–24 months of age) [21–23, 34]. Studies of nusinersen reported mixed results, with the proportion of infants taking full oral nutrition ranging from 84–100% after 19.4–50.1 months of treatment [35, 36].

##### Symptomatic SMA

In patients with Type 1 SMA treated symptomatically, the proportions of patients requiring full or partial nutritional support ranged from 14 to 100% at study endpoint (range of follow-up: 2–48 months) [4, 25]. With nusinersen, over 50% (range: 56–100%) of patients required alternative nutrition at the last study timepoint in all reported studies (range of follow-up: 6–48 months), and the need for tube feeding increased over time in most studies that had longitudinal data (77%, 10/13) [15, 24, 37–40, 43, 44, 46, 47]. With onasemnogene abeparvovec, less than 50% (range:15–46%) of patients required alternative nutrition at the last study timepoint (range of follow-up: 3–24 months) [25, 26] and in six studies with longitudinal results, 50% reported stability in feeding status after treatment initiation. With risdiplam, the proportion of infants able to feed orally was stable over 24 months of treatment; however, there was an increase from baseline in the proportion of patients who received supplemental nutritional support (29% vs. 20%) [28, 55].

Eight observational studies reported oral feeding status in patients with Types 2 and/or Type 3 SMA; six studies reported longitudinal data over a duration of 6.1–38 months. Overall, the proportion of patients reported to require a feeding tube was low in this population. No patients with Type 3 SMA were reported to require feeding support. However, three studies reported rates of tube feeding after treatment to be 5 and 33% in Type 2 SMA [57, 58] and 7.4% in a mixed Types 2/3 SMA population [59] after 3 years, 7.8 months, and 38 months, respectively. In one study of onasemnogene abeparvovec, all patients with Type 2 SMA could feed orally after a median 6.1 months of treatment [60]. Out of five studies reporting longitudinal data for nusinersen, all but one reported that swallowing and feeding ability was stable over the duration of follow-up (range: 6.1–38 months) [57, 58, 61, 62].

Twenty-one publications reported data from populations with a mixed or unclear SMA type, with a follow-up ranging from 6 months to 8 years. In populations treated with nusinersen, the proportions of patients requiring feeding support generally increased over time (range: 6–38 months) [58, 63–67]. With onasemnogene abeparvovec, all publications reported with longitudinal data showed stabilisation or improvement in oral feeding status over 12–33.3 months of treatment [32, 68–72]. No publications reported data from mixed populations treated with risdiplam.

#### Patient-/caregiver-reported outcomes

Fifteen publications from 14 studies were identified that used patient-/caregiver-reported measures to assess swallowing (Table 4).

##### Presymptomatic SMA

The only study in presymptomatic SMA reporting caregiver-reported assessments investigated nusinersen, with swallowing evaluated using the PASA general feeding section. Five years after treatment, 67% of children with two *SMN2* copies, and all children with three *SMN2* copies, were consuming all nutrition by mouth, with 76% of caregivers indicating that they did not have concerns about their child choking during eating [36].

##### Symptomatic SMA

Outcomes amongst children with Type 1 SMA suggest that the magnitude of underlying bulbar deficits at the time of treatment may influence treatment outcomes. In an investigation of the effect of nusinersen on OrSAT scores, Berti et al. found none of the children who relied on a tube for nutrition and tracheostomy for ventilation at baseline (*n* = 4) exhibited improvement in OrSAT score after 18 months of treatment [73]. This was in contrast with children who at baseline solely required a tube for nutrition (no tracheostomy; *n* = 4), who all exhibited numerical improvement in OrSAT score ranging from 1 to 6 points post-nusinersen; however, it is important to note that these improvements were not robust enough to facilitate full oral nutrition in any of the cases. Similarly optimistic results were observed amongst children without the need for tube nutrition at baseline (*n* = 12): these children generally maintained or improved their OrSAT score, and 83% maintained the ability to swallow, with no need for tube feeding after 18 months [73].

Five studies reported patient-/caregiver-reported bulbar outcomes with nusinersen in Types 2 and 3 SMA after 2 months to 3.7 years of treatment. Of four investigations reporting pre- and post-treatment assessments, studies demonstrated that patients generally maintained baseline swallowing integrity. One study reported improvements in swallowing and speech in 5% of patients after 1 year of treatment and in one study a reduction was reported in the proportion of patients being identified by the PASA general feeding score as being tube fed from baseline to 3.7 years of follow-up (2.5% vs. 1.7%) [74].

Similar findings, suggesting maintenance or improvement in bulbar integrity for patients with Type 2 or 3 SMA, were found after 1–2.5 years of treatment with risdiplam [75–78]. Interestingly, the proportion of individuals exhibiting improvement was highly variable within and across studies, ranging from 20 to 100% [75, 78]. This was in part dependent on the outcome used, with nearly twice as many patients from the same study reporting bulbar improvement on the Sydney Swallow Questionnaire than on the Revised Amyotrophic Lateral Sclerosis Functional Rating Scale (47% vs. 83%) [77].

## Discussion

Progressive impairment of swallowing physiology and function has historically been reported amongst patients spanning the SMA severity spectrum [3–6]. These deficits pose substantial risks to respiratory and nutritional health and are of great detriment to quality of life [3, 4]. Despite the significance of swallowing impairments, little is known regarding the effect of DMTs on feeding and swallowing in SMA.

Although we identified 72 studies that evaluated bulbar outcomes after DMTs in this review, nearly all were observational studies, and 32% (23 of 72) of studies did not collect bulbar status both pre- and post-treatment. These limitations significantly restrict our ability to draw conclusions regarding the effects of DMTs on bulbar outcomes. Additionally, the heterogeneity, and often questionable validity of the bulbar outcomes that were used, further convoluted the interpretation of results within and across investigations.

Nearly all investigations evaluated bulbar outcomes using functional swallow outcomes, such as oral intake status, and patient-reported outcomes. Whilst functional swallowing outcomes are of substantial clinical relevance and it is crucial that they are evaluated, they are subject to limitations in their sensitivity as they are dependent on a myriad of personal and clinical variables that may not pertain to a patient’s underlying bulbar integrity. For example, when considering oral feeding status, it is commonly appreciated in the field of dysphagia that recommendations to utilise alternative nutrition such as a gastrostomy tube, implement compensatory strategies such as thickened liquids, or cease oral intake altogether are subjective, and the decision of when to initiate an intervention is highly dependent on the evaluating provider. Additionally, oral feeding status may naturally evolve over time as patients age. Infants with bulbar deficits are more likely to receive alternative nutrition as they age due to the demonstrated persistence of problems throughout a wait-and-see period that eventually warrants intervention from their care team. As these infants continue to age and become less fragile, many caregivers decide to reinitiate some oral feeds for the infant’s enjoyment.

Additionally, patient-reported outcomes are also based on the perception of impairments that rely on an individual comparing their current function to their prior ability. It becomes even more challenging to assess swallowing function in others, particularly in young children who may not be able to articulate what they experience when swallowing.

Consequently, pairing these functional outcomes with more sensitive measures of physiology is critical in fully understanding bulbar integrity. We found that physiological instrumental assessments were rarely utilised within the identified studies, and when they were used, results were not reported using validated metrics. These findings highlight a lack of consensus within the medical community when it comes to the evaluation of bulbar integrity in SMA. Whilst the recent development of functional swallowing outcomes specific to patients with SMA hold promise in filling this gap moving forward [73, 79], true understanding of underlying integrity requires pairing these outcomes with measures of underlying physiology as is routinely done in the evaluation of motor control. Indeed, other recent studies outside of our search parameters have demonstrated that the inclusion of additional physiological measures such as lip and tongue strength and endurance and mouth opening can enable a comprehensive assessment of bulbar function impairments and track more subtle changes in function in SMA [80, 81].

Despite these limitations, our findings suggest that the majority of presymptomatic patients treated with DMTs achieve good swallowing outcomes, with most patients able to achieve full oral nutrition without profound functional deficits. This outcome is further supported by clinical and real-world data published after the data cut of our SLR [82, 83].

These data are encouraging, as previously, without treatment, almost all patients with Type 1 SMA would require exclusive tube feeding due to severe bulbar deficits [8, 84, 85]. Although these outcomes are promising, the aforementioned limitations in methods used to assess bulbar integrity warrant more cautious optimism. Patients consuming full oral nutrition can concurrently have profound swallowing deficits that pose functional limitations for eating efficiency, obtainment of appropriate nutrition, and respiratory health. To have functional swallowing integrity, an individual must not only have full oral nutrition but do so whilst maintaining good respiratory health [25]. Future investigations evaluating the ability of patients to achieve all swallowing endpoints necessary for full function are warranted.

In addition, though the majority of children treated before the onset of symptoms acquired full oral nutrition, not all children achieve these favourable outcomes, with some studies reporting that only 84% of patients achieved full oral nutrition. One theory for the mechanism contributing to this discrepancy is that in infants at risk of developing SMA, there is a clinically silent prodromal phase of disease progression in the late neonatal or early postnatal stage prior to the provision of DMT [86]. The potential for clinically silent, pre-treatment disease progression that may affect outcomes of DMTs warrants further investigation, as it has profound implications for managing caregiver expectations and exploring the safety and effectiveness of extending treatment initiation to the prenatal period [87].

The promising outcomes in patients treated prior to symptom onset are in strong contrast to bulbar outcomes amongst patients treated after symptoms have manifested. Identified studies suggest there is wide variability in bulbar outcomes within this patient population, with the proportion of patients obtaining full oral nutrition ranging from 0 to 86%. It is likely that much of the variability is attributable to differences in pre-treatment status.

Previous research in untreated patients with SMA revealed pathological changes in critical regulators of oropharyngeal swallowing physiology [88, 89]. Outcomes with DMTs are dependent on the composition of the motor neuron pool when treatment is initiated and the extent of the neuronal damage, which are heavily affected by the severity of SMA and disease duration. As such, patients who have deficits in key neural regulators of swallowing at treatment onset are not necessarily anticipated to experience improvements after treatment, but instead, may experience maintenance of baseline function. Results from this review indicate mixed results relating to the ability to maintain bulbar function in patients treated after symptom onset, with variables that influence outcomes including severity of deficits at baseline as well as the type of outcome being reported. Furthermore, patients with profound impairments at baseline, such as those requiring full alternative nutrition, have reached the floor of most assessment scales, and therefore will likely demonstrate maintenance of function at follow-up due to low prognosis for improvement. In contrast, scales are able to capture changes in patients who have more mild–moderate impairments at treatment initiation, such as those who consume some oral nutrition but receive supplemental nutrition by tube.

Although the heterogeneity in bulbar outcomes limited the ability to draw robust conclusions about DMT effects, it is plausible that other treatment-related factors, such as pharmacodynamics and pharmacokinetics, as well as the biodistribution/transduction of a drug within the body, may also influence how bulbar function responds to treatment. Tissue-dependent concentration of nusinersen in autoptic samples from five patients suggested that nusinersen distribution is rostro-caudal, with less distribution to motor neurons in the brainstem than to lumbar and thoracic segments of the spinal cord [90]. In a study by van der Heul et al. in patients treated with nusinersen, this was hypothesised as being implicated in a suboptimal bulbar treatment response [4]. Conversely, both onasemnogene abeparvovec and risdiplam have shown extensive distribution throughout and beyond the central nervous system (CNS), supporting a potentially favourable outcome of these DMTs on bulbar function in SMA. A study examining post-mortem tissue samples after treatment with onasemnogene abeparvovec demonstrated widespread homogeneous distribution of vector genomes and transgenes throughout the CNS, and increased SMN protein levels in the brainstem [91]. Further studies are required more specifically to determine the number and types of cells transduced in these regions, as, although neurons are at least one major target, liver vector genomes demonstrated a 300–1,000-fold increase in transduction over the CNS [91]. In a study by Poirier et al., a homogeneous distribution of risdiplam was observed throughout the CNS, plasma and peripheral tissues in three different animal species, with similar penetration observed in the brain stem and cortex regions via the blood–brain barrier [92]. The distribution is expected to be the same in humans given the same mechanistic rationale [93]. It is important to interpret this biodistribution data with caution as they originate from heterogeneous and limited sources. More research is needed to confirm these observations, but future research should consider this as a possible prognostic factor influencing bulbar function treatment response.

## Conclusions

Our findings suggest:

1. The ability to draw conclusions on the effects of DMTs on swallowing is limited by the use of heterogeneous functional swallowing outcomes across the literature, and that studies frequently did not collect information about pre-treatment bulbar integrity.
2. Patients treated with DMTs prior to symptom onset typically have good feeding and swallowing outcomes.
3. Swallowing and feeding outcomes amongst patients treated with DMTs after symptom onset appear largely dependent on the level of impairment at baseline.
4. It is plausible that other treatment-related factors, such as pharmacodynamics and pharmacokinetics, as well as the biodistribution/transduction of a drug within the body, may also influence how bulbar function responds to treatment; however, more evidence and further observations would be needed to confirm these aspects.

Our findings highlight the need for future investigations to systematically evaluate both physiological and functional aspects of bulbar integrity to allow stronger conclusions to be drawn regarding the effects of DMTs.

## Electronic supplementary material

Below is the link to the electronic supplementary material.

Supplementary Material 1

## Data Availability

All data collected from the SLR can be found within this manuscript document and accompanying supplemental materials.

## Abbreviations

CNS: Central nervous system
DMT: Disease-modifying therapy
PASA: Parent assessment of swallowing ability
PRISMA: Preferred reporting items for systematic reviews and meta-analyses
RCT: Randomised control trial
SLR: Systematic literature review
SMA: Spinal muscular atrophy
SMN: Survival of motor neuron
