# Supplementary material for: Swallowing and feeding after disease-modifying treatment for spinal muscular atrophy: a systematic review of assessment modalities and outcomes
Source: Orphanet J Rare Dis. 2026 Jan 8;21:7. doi: 10.1186/s13023-025-04118-z (PMC12781761; doi:10.1186/s13023-025-04118-z)
Supplement: Supplementary file 1 — Supplementary Material 1 [file 13023_2025_4118_MOESM1_ESM.docx]

**Additional Table 1: Search protocol**

| No. | Query | Results | Facet |
| --- | --- | --- | --- |
| **EMBASE and MedLine** | | | |
| **1** | **'spinal muscular atrophy'/de** | **10,859** | **Disease facet** |
| **2** | 'chronic spinal muscular atrophy':ab,ti OR 'spinal muscle atrophy':ab,ti OR 'spine muscle atrophy':ab,ti OR 'spinal muscular atrophy':ab,ti OR sma:ab,ti OR kennedy:ab,ti OR Kugelberg:ab,ti OR ‘werdnig hoffman’:ab,ti | **54,648** |  |
| **3** | #1 OR #2 | **59,063** |  |
| **4** | 'nusinersen'/exp OR 'Onasemnogene Abeparvovec'/exp OR 'risdiplam'/exp OR 'apitegromab'/exp | **2,650** | **Intervention facet** |
| **5** | 'nusinersen':ab,ti OR 'isis 396443':ab,ti OR 'isis396443':ab,ti OR spinraza:ab,ti OR 'avxs-101':ab,ti OR 'avxs 101':ab,ti OR 'avxs101':ab,ti OR 'ck-2127107':ab,ti OR 'ck-107':ab,ti OR 'ck2127107':ab,ti OR 'ck107':ab,ti OR 'ck 2127107':ab,ti OR 'ck 107':ab,ti OR 'rg7916':ab,ti OR 'ro7034067':ab,ti OR ‘zolgensma’:ab,ti OR ‘risdiplam’:ab,ti OR ‘rg 7916’:ab,ti OR ‘rg7916’:ab,ti OR ‘ro 7034067’:ab,ti OR ‘ro7034067’:ab,ti OR 'apitegromab':ab,ti OR ‘SRK-015’:ab,ti OR reldesemtiv:ab,ti | **1,957** |  |
| **6** | #4 OR #5 | **2,876** |  |
| **7** | #3 AND #6 | **2,423** |  |
| **8** | **#3 AND #6 AND [15-02-2023]/sd NOT [23-02-2024]/sd** | **573** | **Final** |
| **Cochrane search** | | | |
| **1** | **MeSH descriptor: [Muscular Atrophy, Spinal] explode all trees** | **147** | **Disease facet** |
| **2** | “chronic spinal muscular atrophy” OR “spinal muscle atrophy” OR “spine muscle atrophy” OR “spinal muscular atrophy” OR “sma” | **1,183** |  |
| **3** | #1 OR #2 | **1,217** |  |
| **4** | “nusinersen” OR “isis 396443” OR “isis396443” OR “spinraza” OR “zolgensma” | **87** | **Intervention facet** |
| **5** | "avxs-101" OR "avxs 101" OR "avxs101" OR "ck-2127107" OR "ck-107" OR "ck2127107" OR "ck107" OR "ck 2127107" OR "ck 107" OR "rg7916" OR "ro7034067" OR “risdiplam” OR “apitegromab” OR “SRK-015” OR “reldesemtiv” | **111** |  |
| **6** | #4 OR #5 | **184** |  |
| **7** | **#3 AND #6 with Publication Year from 2023-2024** | **18** | **Final** |

**Additional Table 2: PICOS criteria**

| **POPULATION** | Presymptomatic, Types 1, Type 2, or Type 3 SMA |
| --- | --- |
| **INTERVENTION** | Any pharmacological intervention |
| **COMPARATOR** | Any pharmacological intervention |
| **OUTCOMES** | **Efficacy:**  Bulbar outcomes |
| **STUDY DESIGN** | **Inclusion criteria**   - RCTs - Non-RCTs - Single-arm trials - Real-world observational studies (prospective and retrospective)   **Exclusion criteria**   - Cross-sectional studies - Case series/ case reports |

HRQoL, health-related quality of life; PedsQL, Pediatric Quality of Life Inventory; RCT, randomised clinical trial; SMA, spinal muscular atrophy.

**Additional Table 3: Data sources for literature search**

| Bibliographic  databases | - Embase^®^ - MEDLINE^®^ - Cochrane CENTRAL |
| --- | --- |
| Supplementary sources | Conferences   - American Academy of Neurology (AAN) - European Academy of Neurology (EAN) - World Muscle Society (WMS) - European Paediatric Neurology Society (EPNS) - Cure SMA - Annual SMA Research & Clinical Care Meeting - Scientific International Congress on Spinal Muscular Atrophy (SMA Europe) - International Society for Pharmacoeconomics and Outcomes Research (ISPOR; International, Europe, Latin America and the Asia-Pacific) - Academy of Managed Care Pharmacy (AMCP) - European Neurological Society (ENS) - European Paediatric Neurology Society (EPNS) - TREAT-NMD |

NMD, neuromuscular disease; SMA, spinal muscular atrophy
